# Assessment of scatter radiation dose and absorbed doses in eye lens and thyroid gland during digital breast tomosynthesis

**DOI:** 10.1002/acm2.12486

**Published:** 2018-11-24

**Authors:** Thunyarat Chusin, Kosuke Matsubara, Akihiro Takemura, Rena Okubo, Yoshinori Ogawa

**Affiliations:** ^1^ Department of Quantum Medical Technology Division of Health Sciences Graduate School of Medical Science Kanazawa University Kanazawa Japan; ^2^ Department of Radiological Technology Faculty of Allied Health Sciences Naresuan University Phitsanulok Thailand; ^3^ Department of Quantum Medical Technology Faculty of Health Sciences Institute of Medical, Pharmaceutical and Health Sciences Kanazawa University Kanazawa Japan

**Keywords:** digital breast tomosynthesis, eye lens dose, mammography, optically stimulated luminescence dosimeter, scatter radiation

## Abstract

Digital breast tomosynthesis (DBT) is an alternative tool for breast cancer screening; however, the magnitude of peripheral organs dose is not well known. This study aimed to measure scattered dose and estimate organ dose during mammography under conventional (CM) and Tomo (TM) modes in a specific DBT system. Optically stimulated luminescence dosimeters (OSLDs), whose responses were corrected using a parallel‐plate ionization chamber, were pasted on the surface of custom‐made polymethyl methacrylate (PMMA) and RANDO phantoms to measure entrance surface air kerma (ESAK). ESAK measurements were also acquired with a 4.5‐cm thick breast phantom for a standard mammogram. Organ dose conversion factors (CF_D_) were determined as ratio of air kerma at a specific depth to that at the surface for the PMMA phantom and multiplied by the ratio of mass energy absorption coefficients of tissue to air. Normalized eye lens and thyroid gland doses were calculated using the RANDO phantom by multiplying CF_D_ and ESAK values. Maximum variability in OSLD response to scatter radiation from the DBT system was 33% in the W/Rh spectrum and variations in scattered dose distribution were observed between CM and TM. The CF_D_ values for eye lens and thyroid gland ranged between 0.58 to 0.66 and 0.29 to 0.33, respectively. Mean organ doses for two‐view unilateral imaging were 0.24 (CM) and 0.18 (TM) μGy/mAs for the eye lens and 0.24 (CM) and 0.25 (TM) μGy/mAs for the thyroid gland. Higher organ doses were observed during TM compared to CM as the automatic exposure control (AEC) system resulted in greater total mAs values in TM.

## 1 INTRODUCTION

Digital breast tomosynthesis (DBT) is a 3D imaging system that tends to be predominantly used as a diagnostic mammogram for clinical symptoms and as an alternative tool for breast cancer screening.^1^, ^2^ The advantages of DBT have been confirmed by several studies^1^, ^2^, ^3^, ^4^ and include superior early detection of small cancer, lower recall rate, and improved visualization of breast abnormalities. However, radiation risk from mammography is a significant concern among patients, especially among those undergoing routine breast screening, and their physicians.^5^ Therefore, one study by Chetlen et al.^6^ quantified exposure in five organs of interest during a routine digital mammography (DM) procedure and disseminated this information to health care providers. They evaluated scatter doses at the skin surface overlaying these five organs in 207 patients using optically stimulated luminescence dosimeters (OSLDs), and found that the mean doses at the sternum, the thyroid gland, the salivary gland, the eye lens, and the uterus were 870, 245, 200, 25, and 11 μGy, respectively. Recently, another study used Monte‐Carlo simulations on organ doses from a specific DBT system and reports an increase of up to 21% in thyroid gland and 9% in lung (ipsilateral) during DBT acquisition compared to DM acquisition.^7^ However, those organ doses were quantified only from the craniocaudal (CC) view and highly radiosensitive organs such as the eye lens were not considered.

The DBT system provides dual acquisition modes based on DM or DBT acquisitions as there are geometric differences in the acquisition setup between these two modes. Specifically, while the x‐ray tube rotates across a compressed breast within a limited angle range during DBT acquisition, it remains fixed during DM acquisition,^8^ and the absorbed dose at each acquisition mode also varies when used with the automatic exposure control (AEC) system.^7^, ^9^ These differences should be taken into account for estimating scattered dose at organs of interest during DBT imaging. Currently, OSLDs play an importance role in point dose measurement during both radiotherapy and diagnostic imaging due to their characteristics such as high sensitivity, small size, tissue equivalence density, and reusability. While McKeever et al.^10^ have reported that OSLDs are capable of measuring doses as low as 10 μGy, linear response among commercially available OSLDs starts at approximately 50 μGy. In addition, previous studies^11^, ^12^ have demonstrated the feasibility of using commercial OSLDs in the mammography energy range by adding a correction factor.

Thus, this study aimed to measure and compare scatter doses between DM and DBT acquisitions from a specific DBT system and to estimate absorbed doses in the eye lens and the thyroid gland during two‐view mammography under both DM and DBT modes. We provide data on scattered doses and organ doses and discuss  variations in these doses and their potential radiation risk during DM and DBT acquisitions in clinical scenarios.

## 2 MATERIALS AND METHODS

A DBT system (MAMMOMAT Inspiration; Siemens Medical Solutions Inc., Erlangen, Germany) with dual acquisition modes for DM and DBT was used. DM acquisition was used in the conventional mode (CM) while the DBT acquisition was specified as Tomo mode (TM) which acquires 25 projections of breast tissue from different tube angles between −25° and +25° at 2° intervals.

### 2.A Radiation dosimeter and correction

**Figure 1:**
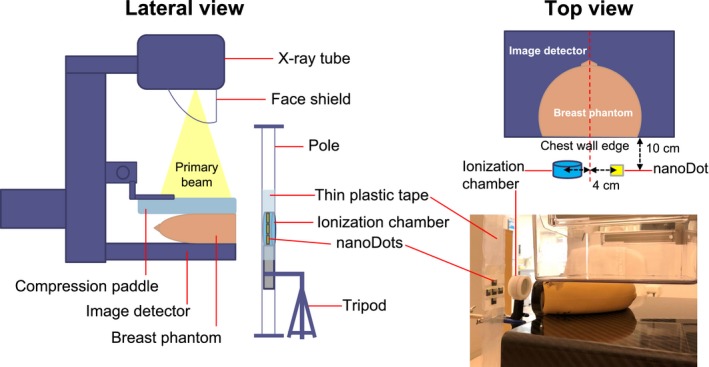
The experimental setup for correcting the nanoDots with an ionization chamber for use under conditions encountered during scatter dose measurement.

The nanoDot OSLD (Landauer Inc., Glenwood, IL, USA) is a small disk made from carbon‐doped aluminum oxide (Al_2_O_3_:C) that is enclosed within a light tight plastic frame and measures 10 × 10 × 2 mm. It was used in combination with the microStar reader (Landauer Inc.) for all dose measurements. The lower limit of detection of this OSLD system is 46.7 μGy, according to the calibration certificate provided by the manufacturer. NanoDots were corrected using a parallel‐plate ionization chamber (Radcal Corp., Monrovia, CA, USA) for use under specific conditions encountered in our DBT system while measuring scatter radiation generated during mammography. Figure 1 illustrates the experimental setup for nanoDot correction. A 4.5 cm thick breast phantom (CIRS Inc., Norfolk, VA, USA) with an average glandular tissue composition of 50% was positioned for CC view at the center of the image detector with a compression paddle. Five nanoDots were pasted using thin plastic tape and then attached to a pole such that they were similarly placed in air. The ionization chamber was attached to a tripod, and the nanoDots and the ionization chamber were placed symmetrically and laterally at the side of chest wall edge, 4 cm from the center of image detector and 10 cm away from the chest wall edge, such that they were positioned mid‐level to the breast phantom (Fig. 1).

The exposure parameters were manually set for the tungsten target and the rhodium filter combinations (W/Rh) with tube voltages of 27, 28, and 29 kV and a tube current‐time product of 160 mAs. The breast phantom was irradiated six times at each exposure setting under CM operation and the scatter dose was allowed to accumulate in the nanoDots for the six irradiations. The nanoDots and the ionization chamber simultaneously recorded the amount of scatter radiation generated predominantly from the irradiated breast phantom. NanoDot correction was repeated twice using a new set of five nanoDots, and finally, all exposed nanoDots were read consecutively three times along with three control nanoDots in the microStar reader to reduce measurement uncertainty; the average of the three readings from each nanoDot was used for calculating the correction factor of the nanoDot (CF_OSLD_) as

(1) $${\mathrm{CF}}_{\mathrm{OSLD}}=\frac{K_{\mathrm{IC}}}{K_{\mathrm{OSLD}}}-\mathrm{BG}$$

where $K_{\mathrm{IC}}$ is average air kerma from the ionization chamber (mGy), *K*_OSLD_ is average air kerma readout from the five nanoDots (mGy), and BG is average background readout from the control nanoDots (mGy).

### 2.B Entrance surface air kerma measurements

**Figure 2:**
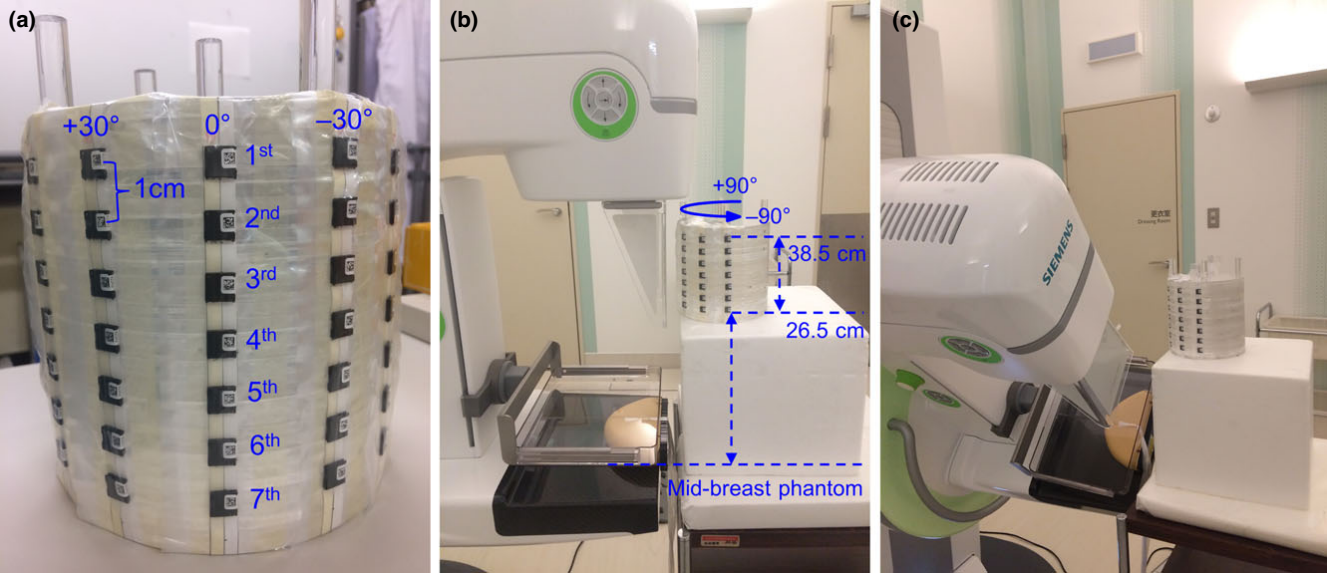
NanoDot positions onto custom‐made PMMA phantom at various locations to assess differences in scatter dose received according to angles and distances (a). Scatter radiation dose could be detected from angles of −90° to +90° and at distance ranging from 26.5 to 38.5 cm, depending on the experimental setting; CC view acquisition (b), MLO view acquisition (c).

Entrance surface air kerma (ESAK) was measured to characterize scatter dose during CM and TM. To simulate a patient's head, we used a custom‐made, polymethyl methacrylate (PMMA) cylindrical phantom that was 16 cm in height and diameter. Figure 2(a) illustrates nanoDot placements on the PMMA phantom; 49 nanoDots were pasted on surface of the PMMA phantom such that they covered angles of −90°, −60°, −30°, 0°, +30°, +60°, +90°. These angles were used based on locations that receive scatter radiation. Each placement angle group consisted of seven nanoDots placed along the vertical direction of the phantom with a gap of 1 cm between successive nanoDots. The fourth nanoDot was marked as 0° and corresponded to the center point on the PMMA phantom. The breast phantom was positioned at the center of image detector along with the compression paddle and face shield for CC and mediolateral oblique (MLO) view acquisitions. The PMMA phantom was positioned on a polystyrene box such that the center point was located at a vertical height of 32.5 cm (a surrogate of eye level of a female RANDO phantom) from the center the breast phantom and horizontal to the mid‐breast phantom, with the phantom facing as close as possible to the edge of the chest wall [Fig. 2(b)]. This experimental setting enabled the nanoDots receive scatter radiation from angles ranging from −90° to +90° with receiving distances varying from 26.5 to 38.5 cm, based on the locations of nanoDots [Fig. 2(b)].

The exposure parameters were set for a W/Rh combination of 28 kV and automatic tube current‐time product, and this setting was chosen for ESAK measurements because, according to manufacturer's specification, only the W/Rh combination could be modified in TM mode and also because the AEC system identified a tube voltage of 28 kV as optimal for the 4.5 cm compressed breast phantom. All ESAK measurements were performed in both CM and TM by nanoDots and the nanoDots were irradiated thrice during each measurement to increase the amount of scattered dose received. All nanoDots were read thrice along with the three control nanoDots (for measurement of background radiation). The actual scatter dose, in terms of ESAK, was obtained by subtracting background radiation, dividing them by three, and then correcting them based on the CF_OSLD_ value for the W/Rh 28 kV spectrum.

### 2.C Organ dose conversion factors

**Figure 3:**
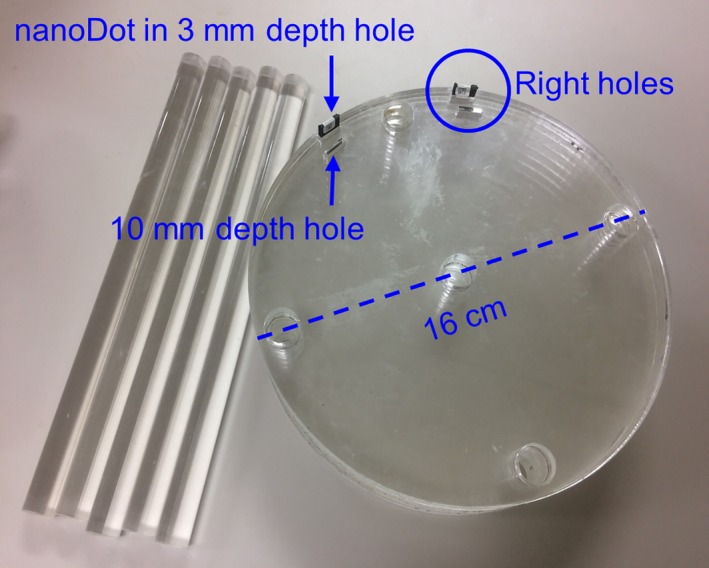
The custom‐made PMMA phantom used for air kerma measurement.

The organ dose conversion factor (CF_D_) for the W/Rh 28 kV spectrum was calculated to estimate absorbed doses at organs that received scatter radiation during the mammogram under both CM and TM acquisitions. The nanoDots were inserted into left‐ and right‐sided holes at specific depths in the PMMA phantom (Fig. 3); specifically, two nanoDots each at a depth of 3 mm and two nanoDots at a depth of 10 mm. Other two nanoDots were pasted onto corresponding locations on the surface of the PMMA phantom.

The positions of the breast and PMMA phantoms and the exposure parameters were identical to ESAK measurements (as described in Section 2.B). Air kerma at the specified depths and the surface were measured separately for the CC and the MLO view acquisitions and in both CM and TM. The air kerma from all exposed nanoDots was measured by reading them thrice and using background subtraction. The CF_D_ was defined as the ratio of air kerma at corresponding depths to surface, and the ratio of mass energy absorption coefficients of tissue to air, and was quantified using the following expression

(2) $${\mathrm{CF}}_{D}=\frac{K_{\mathrm{depth}}}{K_{\mathrm{surface}}}\times \frac{{\left(\frac{\mu_{\mathrm{em}}}{\rho }\right)}_{\mathrm{tissue}}}{{\left(\frac{\mu_{\mathrm{en}}}{\rho }\right)}_{\mathrm{air}}}$$

where *K*_*depth*_ is average air kerma (mGy) at a specific depth, $K_{\mathrm{surface}}$ is average air kerma (mGy) at the surface, ${\left(\frac{\mu_{\mathrm{en}}}{\rho }\right)}_{\mathrm{tissue}}$is mass energy absorption coefficient of the eye lens and soft tissue (cm^2^/g), and ${\left(\frac{\mu_{\mathrm{en}}}{\rho }\right)}_{\mathrm{air}}$ is mass energy absorption coefficient of air (cm^2^/g). The mass energy absorption coefficients were obtained from the National Institute of Standard and Technology (NIST),^13^ according to the effective energy of primary beam, which was estimated using the half value layer of the W/Rh 28 kV spectrum.

### 2.D Organ dose estimation

**Figure 4:**
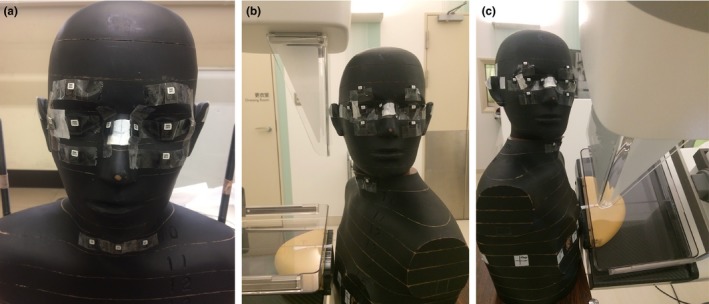
Locations of the nanoDots when pasted onto the surface of the RANDO phantom (a). The RANDO phantom and breast phantom on CC (b) and MLO (c) views, positioned with the compression paddle and face shield.

The absorbed doses at the eye lens and the thyroid gland were estimated using the RANDO phantom. A set of 13 nanoDots were pasted on skin of a female RANDO phantom (Phantom Laboratory, Salem, NY, USA) with 10 nanoDots around the eyes and three nanoDots around the thyroid gland, as shown in Fig. 4(a). Three other nanoDots were used as controls for measuring background radiation. The RANDO phantom and the breast phantom were positioned similar to a patient positioned for CC or MLO views [Figs. 4(b) and 4(c)]. The x‐ray tube gantry was set at 45° projection in the MLO view. The cranial RANDO phantom was rotated toward the contralateral side for both CC and MLO views.

Entrance surface air kerma measurements were acquired using four sets of nanoDots for the CC and the MLO views in CM and TM, with a tube voltage of 28 kV and W/Rh combination and automatic tube current‐time product settings that were identical to those used for previous ESAK measurements. Three irradiations were conducted for each measurement to allow sufficient accumulation of radiation in the nanoDots, and average of three readout values from each nanoDot, after background subtraction, was divided by three. The ESAK obtained using the RANDO phantom was used to estimate eye lens and thyroid gland doses using the following equation:

(3) $$D=\mathrm{ESAK}\times {\mathrm{CF}}_{\mathrm{OSLD}}\times {\mathrm{CF}}_{D}$$

where ESAK is the average air kerma readout from the nanoDots, CF_OSLD_ is the correction factor of the nanoDots, and CF_D_ is the organ dose conversion factor.

## 3 RESULTS

### 3.A NanoDot correction

Scattered radiation sensed by the nanoDots was different from those of the reference dosimeter by 11.9%–32.5%. The reproducibility of scattered dose measurement using nanoDots had a coefficient of variation (CV) of less than 8.7%, as estimated from two measurements. The cumulative scattered dose from six irradiations, CF_OSLD_, and CV values are listed in Table 1. The CF_OSLD_ at 28 kV was applied for all readout values obtained from the nanoDots.

**Table 1:** Correction factor values for the nanoDots (CF_OSLD_) during scatter radiation measurement

Target/Filter	Tube voltage (kV)	Exposure (mAs)	Mean cumulative dose (μGy)	CF_OSLD_ (SD)	Coefficient of variation (%)
W/Rh	27	160	120.8	0.77 (0.07)	8.7
	28	160	127.0	0.89 (0.00)	0.2
	29	160	160.2	0.80 (0.01)	1.0

SD, standard deviation.

### 3.B ESAK measurements

**Figure 5:**
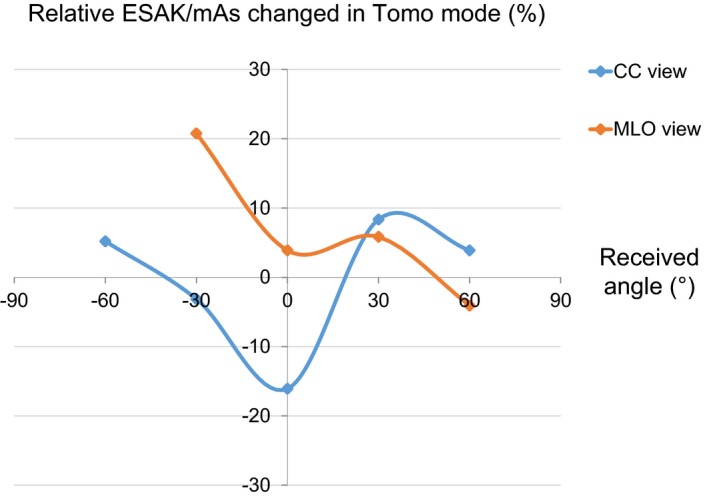
The percent differences in ESAK per mAs between conventional (CM) and Tomo modes (TM) as a function of receiving angles.

All nanoDot cumulative doses of less than 50 μGy after three irradiations were excluded from analysis in this study. The measured ESAK values in the PMMA phantom are provided in Table 2 and Fig. 5 shows percentage differences in ESAK per mAs, averaged for values at identical received distances between TM and CM. Significant differences in ESAK during TM were observed between angles of −60° and +60°. The ESAK during TM at 0° angle was lower by a maximum of 16.1% whereas it increased at angles beyond 0° by a maximum of 20.8%. In the MLO view, ESAK was higher at an angle of −30° compared to +30° due to the effects of primary beam projecting from that side of the phantom.

**Table 2:** The measured ESAK in PMMA phantom

Image acquisition mode	Target/filter	Tube voltage (kV)	Exposure (mAs)	ESAK (μGy)	
				CC view	MLO view
Conventional	W/Rh	28	133.6 ± 1.5	32.3 (16.4, 67.2)	28.0 (16.7, 47.9)
Tomo	W/Rh	28	199.3 ± 18.7	42.4 (16.0, 91.1)	33.9 (17.0, 62.8)

Mean ±SD, Mean (minimum, maximum).

### 3.C Organ dose conversion factors

Table 3 shows CF_D_ values specific for the 4.5 cm compressed breast phantom at the W/Rh 28 kV x‐ray spectrum whose effective energy of the primary beam was 18.4 keV. The effective energy was calculated using the half value layer of 0.56 mmAl of the W/Rh 28 kV spectrum and NIST data. With regard to the mass energy absorption coefficient of the eye lens and the soft tissue, CF_D_ values were used for calculating absorbed doses at depths of 3 and 10 mm from the surface around the patient's head.

**Table 3:** The organ dose conversion factors (CF_D_) for the eye lens and the thyroid gland

Image acquisition	Conventional mode		Tomo mode	
	Eye lens	Thyroid gland	Eye lens	Thyroid gland
CC view	0.66 (0.04)	0.29 (0.01)	0.59 (0.00)	0.32 (0.02)
MLO view	0.59 (0.06)	0.33 (0.03)	0.58 (0.04)	0.30 (0.00)

Mean (SE).

### 3.D Organ doses

In the RANDO phantom study, negligible radiation doses were recorded at the eye lens in the contralateral imaging side during both CM and TM. The absorbed doses at the thyroid gland and eye lens on the imaging side are shown in Table 4. The organ doses tended to be higher during TM compared to CM, because of higher total mAs values observed during TM under AEC operation. Organ dose values per unit of mAs at the eye lens, estimated during two‐view unilateral mammography, were 0.24 and 0.18 μGy/mAs for CM and TM respectively, whereas those for the thyroid gland were 0.24 and 0.25 μGy/mAs for CM and TM, respectively. We found that the dose per mAs for the eye lens decreased by 30.4% in TM compared to CM, whereas in the thyroid gland, it increased slightly by 4.3%.

**Table 4:** The eye lens and thyroid gland doses recorded during two‐view unilateral mammogram in a specific DBT system

Image acquisition mode	Tube voltage (kV)	Exposure (mAs)	Eye lens dose (μGy)	Thyroid gland dose (μGy)
Conventional	28	112.4 ± 15.3	27.1 ± 4.6	26.9 ± 2.9
Tomo	28	160.9 ± 21.4	28.5 ± 5.0	40.2 ± 5.3

Mean ± SD.

## 4 DISCUSSION

We show that the nanoDot response to scatter radiation had a maximum variation of 33% compared to the ionization chamber. This may be explained by the energy dependence of the nanoDot in the energy range utilized during mammography; this value has been reported as 10% in the study by Kawaguchi et al.,^12^ whereas it was 50% in the study by Okazaki et al.^14^ Specifically, Okazaki et al. have reported that the relative responses of nanoDots with CdTe detector at low energy were 0.46 (8.1 keV), 0.87 (16.9 keV), and 1.00 (31.6 keV). Therefore, nanoDots should ideally be corrected for their energy dependence at doses used in mammography using a reference dosimeter for more accurate measurement.

We found that the differences in acquisition geometry between CM and TM had an impact on scattered dose in the PMMA phantom as TM acquisition involved movement of the x‐ray tube over the breast phantom which led to distribution of scatter radiation across all projection angles of the incident beam. This led to lower ESAK per mAs values during TM at a receiving angle of 0° but yielded higher values at receiving angles greater than 0°. Changes in ESAK values in the CC view at receiving angles of −30° and +30° can be explained by inaccurate nanoDot placement, while similar changes in the MLO view are attributable to acquisition geometry as the x‐ray tube gantry was set for oblique projection.

We observed that eye lens dose during TM increased due to greater number of image acquisitions, i.e., 25 projections acquired with a scan time of 25 s;^15^ this caused an increase in the tube current‐time product in the AEC system. Nonetheless, the difference in eye lens dose in the Rando phantom was less than 5.0% between TM (28.5 μGy) and CM (27.1 μGy) even though the total mAs value in TM (160.9 mAs) was 35.5% higher than in CM (112.4 mAs). It is also possible that the number of scattered photons from the breast phantom that reach the eyes were restricted by the energy of the scattered photon. In contrast, absorbed dose in the thyroid gland displayed a large difference of 39.7% between TM (40.2 μGy) and CM (26.9 μGy), possibly because of its anatomically close location to the breast phantom.

Small discrepancies in ESAK values are observable between our study and the study by Chetlen et al.^6^ and can be accounted for by differences in breast phantom thickness and composition between the patient population and the breast phantom*,* and due to nanoDot placements. Chetlen et al.^6^ have reported mean breast thickness and average glandular dose (AGD) in their study population to be 6.1 and 1.36 mGy per view, respectively, while mean ESAK recorded by one nanoDot pasted at the bridge of the nose (a surrogate of eye lens) during DM in the patient population was 25 μGy in two‐view bilateral mammography. Importantly, even though the mean ESAK was less than lower detection limit of the nanoDot (33.5 μGy) in their study, ESAK at the bridge of the nose was highest at 121 μGy. In our study, one of the RANDO phantom eyes recorded a higher the mean ESAK (about 86.4 μGy) and the maximum ESAK value was 131.9 μGy during two‐view bilateral mammography in CM; essentially, eyes close to the imaged side received a higher dose whereas the contralateral eye received a negligible dose, implying that nanoDots should be pasted on eyes that are close to imaged tissue for improving accuracy of measurement. The positions of nanoDots at thyroid gland in the study by Chetlen et al.^6^ are comparable to those employed by us, as they also pasted two nanoDots on the patient's skin over the right and the left thyroid lobes. They report mean ESAK of 245 μGy for the thyroid gland which is similar to our value of 174.9 μGy when differences in compressed breast thickness are taken into account (6.1 vs. 4.5 cm).

On the other hand, large discrepancies in organ doses are seen between values obtained by direct measurement in our study and those obtained by the Monte‐Carlo simulation in other studies. Baptista et al.^7^ have reported thyroid gland doses of 273 μGy for two CC views during DM acquisition and 347 μGy during DBT acquisition; these are almost ten times higher than those measured in our study (29.7 μGy in CM and 47.7 μGy in TM). Procedural variations can account for this difference as the other studies used a female voxel phantom, a computational phantom based on segmented images of a 43‐year‐old patient, and assumed an AGD of 2 mGy for a W/Rh 28 kV x‐ray spectrum for the imaged breast. Thyroid gland doses were calculated relative to the AGD in DM and DBT acquisitions wherein the irradiated beam was simulated for 25 projections at an angular range between −24° and +24° during DBT. Additionally, data on either breast thickness or compression of the phantom were absent in these reports. These details are crucial as, if the phantom breast was not compressed, it may generate more scatter radiation from the breast and result in higher absorbed dose in the peripheral organs. However, they report an increasing trend in thyroid gland dose during tomosynthesis acquisition which is consistent with the results from our study. Sechopoulos et al.^16^ have also used a computational phantom (Cristy phantom) after modifying its elemental composition and tissue densities, and addition of eyes, eye lens, and sternum to the phantom. They used a model that incorporated a compressed breast with 50% glandularity in both CC (5.2 cm) and MLO (5.7 cm) views and assumed glandular doses of 2.0 and 2.5 mGy for the CC and MLO views, respectively. Further, eye lens dose was normalized to breast glandular dose, which then yielded a maximum eye lens dose of 4.4 μGy for two‐view unilateral mammography using the Rh/Rh 35 kV x‐ray spectrum. This value is nearly six times lower than that reported here (27.1 μGy in CM). These discrepancies in scattered dose values between direct measurement and Monte‐Carlo simulations can be accounted for by variations in phantom characteristics and mammographic positioning associated with using a real phantom versus computational phantoms. Authors of the previous studies, namely, Baptista and Sechopoulos, concur that computational phantoms have limitations in their ability to describe the complex shape encountered in real organs (3D shape) and that such phantoms may deviate from the body habitus of patients. Therefore, using such computational phantoms can potentially introduce significant error in estimated organ dose.^16^ Additionally, in study by Castellano et al.,^17^ discrepancies of about 38% in estimated dose are seen between the Cristy phantom and the voxel base phantom, and estimated absorbed dose values at the left lung (ipsilateral) reported by the studies of Baptista^7^ and Sechopoulos^16^ are 385 and 4.4 μGy respectively, even though assumed AGD was similar in both studies.

Our results definitively show that exposure of the eye lens and the thyroid gland cannot be avoided during mammography, either in CM or TM, despite the presence of a face shield. Nevertheless, these doses were in the μGy range which is extremely small and seems negligible when compared to the fact that the current threshold dose for a significant risk to organs such as the lens is 0.5 Gy, as suggested by the International Commission on Radiological Protection (ICRP).^18^ Further, the thyroid gland is considered less radiosensitive compared to the eye lens.

The above notwithstanding, there are limitations to this study. We used a specific breast phantom with breast thickness of 4.5 cm and breast composition of 50% glandular tissue and 50% adipose tissue, and a defined x‐ray spectrum, which is not completely compatible with the clinical scenario where women who undergo mammography have various breast thicknesses and densities. These factors lead to the use of a variety of x‐ray spectra for mammography. For instance, a higher energy spectrum is usually used for thick breasts than that used for the average breast; this may lead to an increase in scattered dose at peripheral organs. Moreover, uncertainty in our results may have occurred due to the angular dependence of nanoDots as we report about 5% difference in scatter radiation compared to Monte‐Carlo simulation‐based measurements by Okazaki et al.^14^

## 5 CONCLUSION

Entrance surface air kerma measurements using OSLDs in a specific DBT system were compared between TM and CM and the observed variation in ESAK values between CM and TM was due to rotation of the x‐ray tube during tomosynthesis acquisition. Organ doses were normalized by multiplying ESAK measured in the Rando phantom and estimated conversion factors. Eye lens dose per mAs was lower by 30.4% whereas thyroid gland dose per mAs was higher by 4.3% in TM compared to CM. Organ doses estimated during two‐view unilateral imaging were higher in TM compared to CM due to greater number of image acquisitions in TM; these led to a higher total mAs value under AEC operation.

## CONFLICT OF INTEREST

We would like to declare that Dr. Ikuo Kobayashi of Nagase Landauer Ltd. provided us the PMMA phantom.
